# Spindle Cell Cardiac Lipoma in an Adolescent: A Case Report and Systematic Review of Cases

**DOI:** 10.1016/j.jaccas.2026.108811

**Published:** 2026-06-19

**Authors:** Si Ling Soh, Ian J.Y. Wee, Rowina Lynne Murray Binti Jeffery Murray, Sivakumar Sivalingam, Aslannif Roslan

**Affiliations:** a Department of Cardiology, National Heart Institute, Kuala Lumpur, Malaysia; b Health Services Research Unit, Singapore General Hospital, Singapore, Singapore; c Department of Cardiology, Sunway Medical Centre, Kuala Lumpur, Malaysia

**Keywords:** cardiac magnetic resonance, cardiac lipoma, echocardiography, imaging

## Abstract

**Background:**

Spindle cell lipoma is a benign adipocytic neoplasm that typically arises in subcutaneous soft tissues and is exceptionally rare in the heart.

**Case Summary:**

A 13-year-old male patient was referred for evaluation of a cardiac murmur. Transthoracic echocardiography demonstrated multiple intracardiac masses involving the aortic valve, interventricular septum, and posterior left ventricular wall, resulting in severe aortic regurgitation and moderate aortic stenosis. Cardiac magnetic resonance imaging revealed a large lobulated mass arising from the left coronary cusp of the aortic valve with extension into the left ventricular outflow tract. Given diagnostic uncertainty, surgical resection with mechanical aortic valve replacement was performed. Histopathology confirmed a spindle cell lipoma. Recovery was uncomplicated, with no residual mass on follow-up imaging.

**Discussion:**

A systematic review identified only 2 previously reported adult cases, both involving cardiac valves. Imaging was nonspecific, and definitive diagnosis relied on histopathology.

**Take-Home Message:**

Spindle cell cardiac lipoma is a rare but clinically significant cause of valvular cardiac masses; histopathological confirmation is essential, and surgical excision appears curative.

## Graphical Abstract

**Figure:**
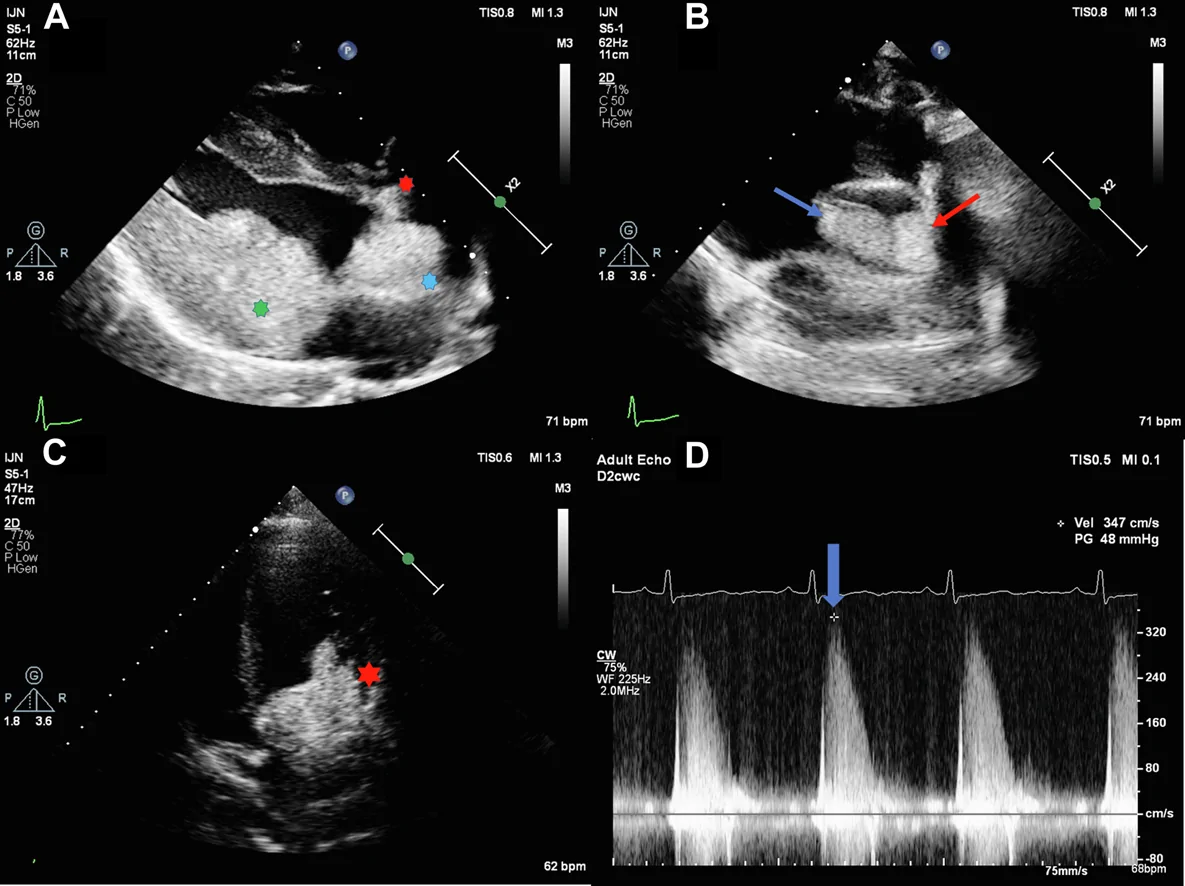

## History of Presentation

A 13-year-old male patient was referred for evaluation of a newly detected cardiac murmur. He was otherwise asymptomatic, with no history of chest pain, syncope, or exercise intolerance. Physical examination revealed an ejection systolic murmur best heard at the left upper parasternal region.

**Take-Home Messages**

• A spindle cell cardiac lipoma is an exceptionally rare benign tumor that may present with clinically significant valvular obstruction.
• Multimodality imaging is useful for detection, but histopathological examination is essential for definitive diagnosis.
• Complete surgical excision appears curative, with excellent short-term outcomes reported to date.

## Past Medical History

There was no significant past medical history. He was not on regular medications, and there was no relevant family history of congenital or acquired cardiac disease.

## Differential Diagnosis

The differential diagnosis for a valvular cardiac mass in a pediatric patient included papillary fibroelastoma, cardiac myxoma, thrombus, lipomatous tumor, and primary cardiac sarcoma.

## Investigations

**Figure 1:**
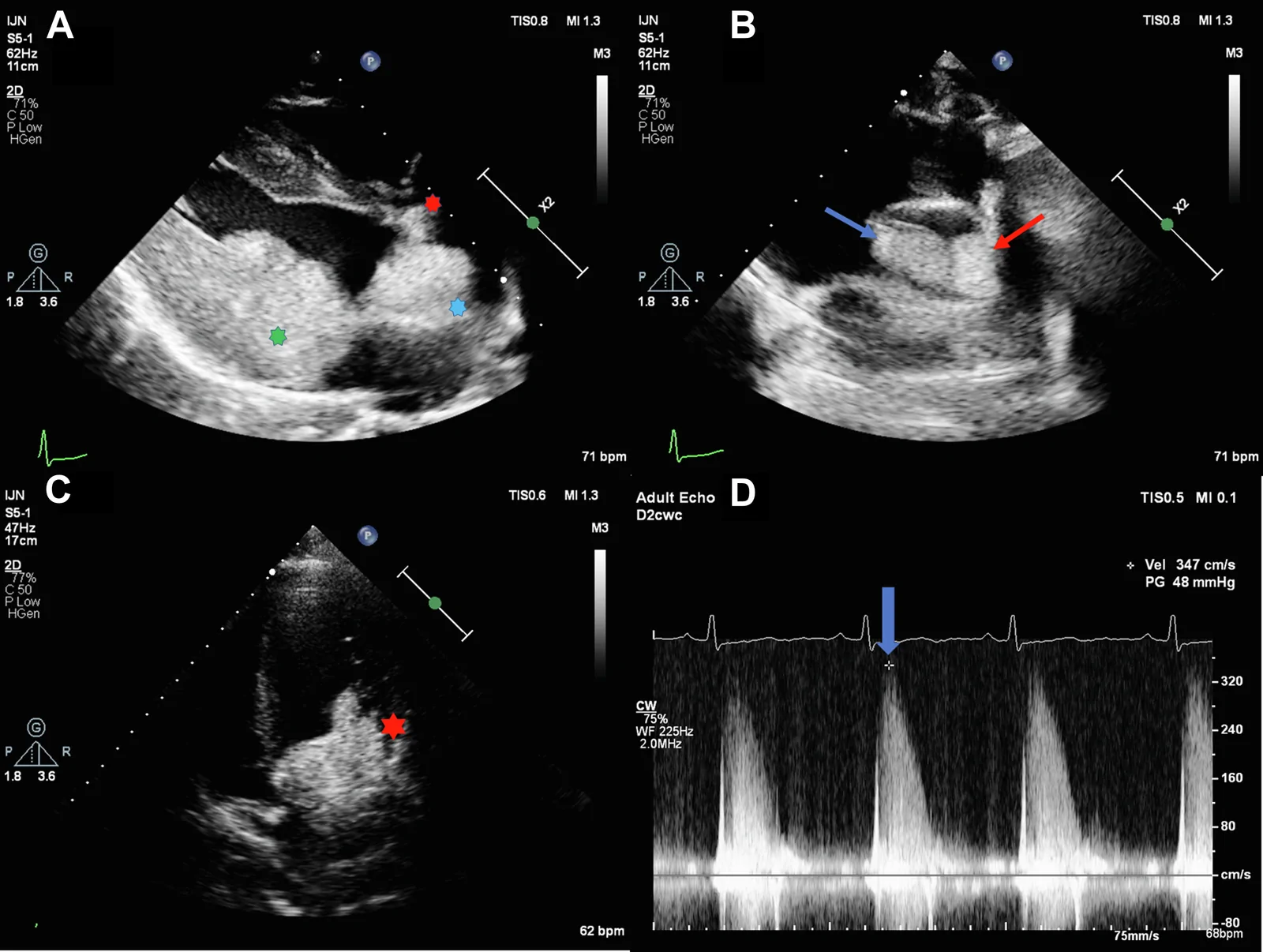
2D Transthoracic Echocardiography (TTE) and Continuous Wave (CW) Doppler Across Aortic Valve (A) Three cardiac masses at the (1) left ventricle wall (green star), (2) noncoronary cusp (blue star), and (3) left coronary cusp (red star) are demonstrated on a parasternal long-axis view in transthoracic echocardiography. (B) Noncoronary cusp (blue arrow) and left-coronary cusp (red arrow) masses seen on a short-axis view in transthoracic echocardiography. (C) Left ventricular wall mass seen on an apical four-chamber view. (D) Doppler signal showing severe aortic regurgitation. bpm = beats/minute; CW = continuous wave; IJN = internal jugular vein; MI = myocardial infarction; PG = pressure gradient; TIS = tissue imaging system; VEL = velocity.

Transthoracic echocardiography (TTE) showed 3 masses—2 attached to the aortic valve (AV) (Figure 1A) with near-complete involvement of the noncoronary and left coronary cusps (LCCs) (2.06 × 1.65 cm) (Figure 1B, Video 1), as well as one additional mass along the left ventricular outflow tract (LVOT) extending to the anterior left ventricle (LV) wall (Figure 1C, Video 2). These resulted in severe aortic regurgitation (AR) and moderate functional aortic stenosis (Figure 1D).

**Figure 2:**
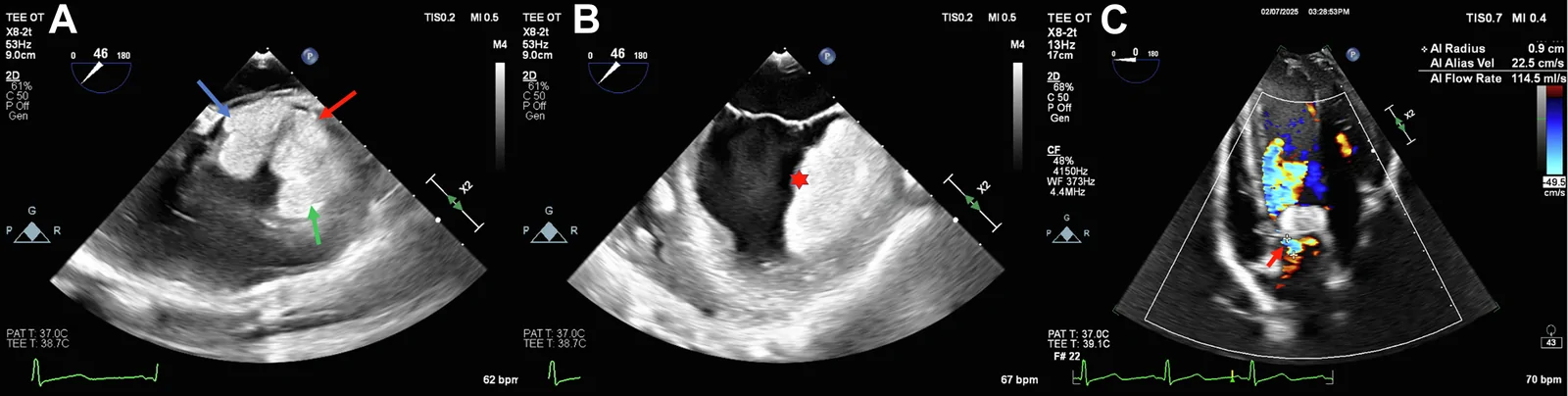
2D Transesophageal Echocardiography (TEE) and Color Flow Doppler Across Aortic Valve (A) Transesophageal echocardiogram mid-esophageal short-axis view showing masses at the noncoronary cusp (blue arrow), left coronary cusp (red arrow), and left ventricular wall mass (green arrow). (B) Mid-esophageal view demonstrating left ventricular wall mass. (C) Color Doppler from a deep transgastric view showing severe aortic regurgitation. AI = aortic insufficiency; CF = colour flow; MI = myocardial infarction; PAT = patient's temperature; TEE OT = transesophageal echocardiography operating theater; TIS = tissue imaging system.

These masses were identified on a transesophageal echocardiogram, which clearly demonstrated the 2 AV masses as well as the third mass extending to the anterior LV wall (Video 3). These findings were further corroborated by 3-dimensional imaging (Video 4). AR was quantified from the deep transgastric view (Figure 2).

Cardiac magnetic resonance imaging was performed for tissue characterization, demonstrating a large, lobulated mass predominantly involving the LCC of the AV. The mass encases the entire leaflet and moves synchronously with AV opening and closure (Videos 5-7). There is marked flow acceleration across the valve, consistent with moderate–severe stenosis, with a peak aortic velocity of 3.5 m/s, along with concomitant moderate AR. Phase-contrast flow mapping quantified a regurgitant volume of 52 mL and a regurgitant fraction of 40%. There is no evidence of extension beyond the valve apparatus or invasion into the adjacent myocardium. On tissue characterization, the AV mass is mildly hyperintense on T1-weighted imaging (1,446 ms), isointense on T2-weighted imaging (60 ms) and demonstrates mild heterogeneous enhancement on rest perfusion as well as on early and late post-gadolinium imaging.

**Figure 3:**
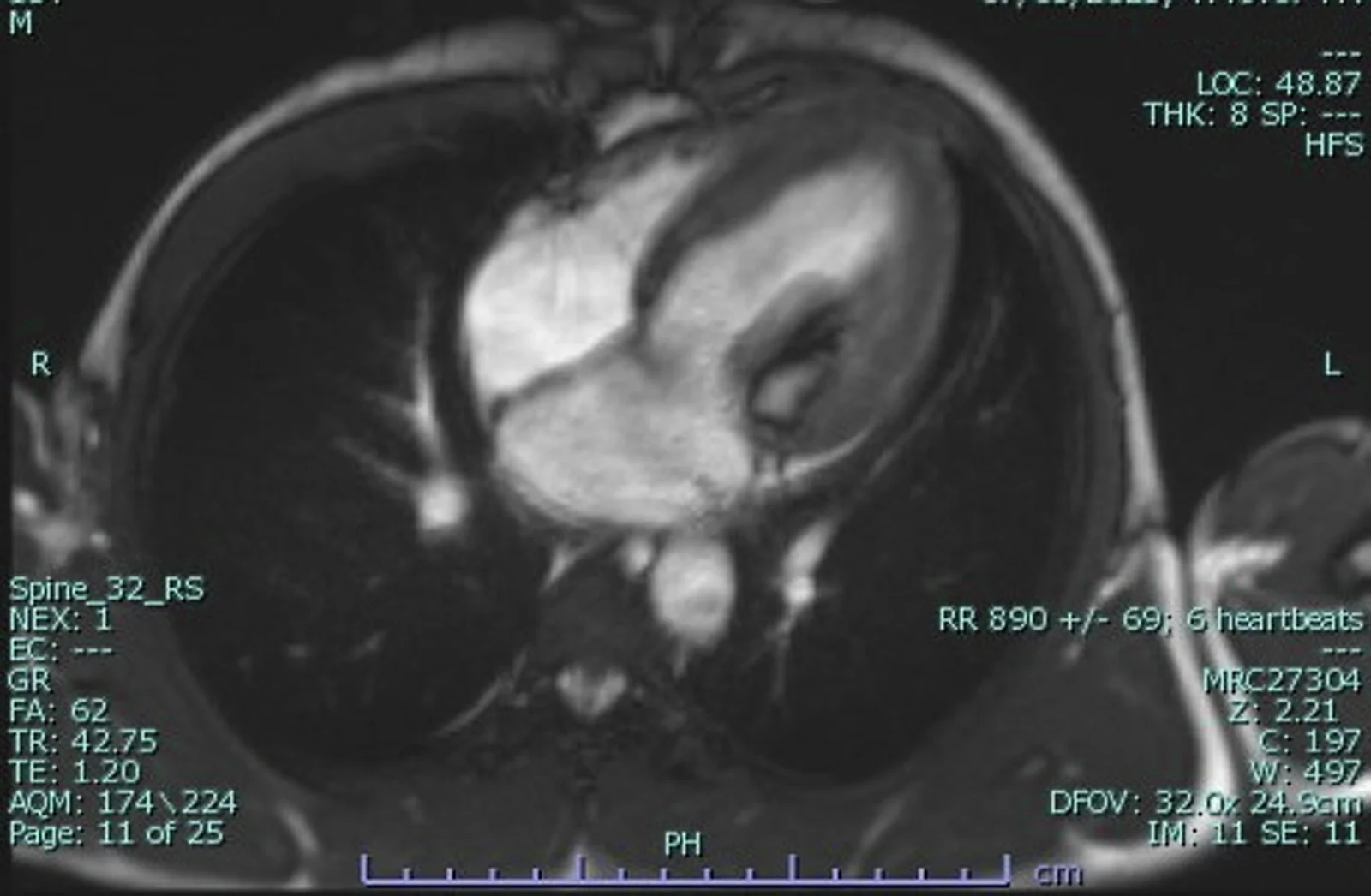
Cardiac Magnetic Resonance Imaging Showing Left Ventricular Outflow Tract Mass Extending into the Basal Anterior Wall of the Left Ventricle, Showing Homogeneous Late Gadolinium Enhancement AQM = acquisition matrix; DFOV = display field of view; EC = echo collection/echo count; FA = flip angle; GR = gradient recall (gradient echo); IM = image; LOC = location/localizer; NEX = number of excitations; PH = potential of hydrogen; RR = reconstruction radius/reconstruction region; TE = echo time; THK = slice thickness; TR = repetition time.

The mass arising from the LVOT is located beneath or adjacent to the AV on the LCC side, extending into the basal anterior wall of the LV (Figure 3). It measures 28 × 20 mm in the LVOT view and 27 × 18 mm in the two-chamber view. This LVOT mass appears hyperintense on both T1-weighted (256 ms) and T2-weighted (70 ms) sequences, shows homogeneous enhancement on first-pass perfusion, and exhibits avid, homogeneous late gadolinium enhancement with nonenhancing peripheral margins. Right ventricular size and function are normal, with no myocardial edema, inflammation, or fibrosis.

## Management

Given the hemodynamic significance of the valvular involvement and the uncertainty of the underlying pathology, the patient underwent surgical resection of the cardiac masses. Intraoperatively, the tumor was noted to involve the AV, necessitating mechanical AV replacement with a 23-mm St Jude Regent prosthesis (Abbott).

## Outcome and Follow-Up

**Figure 4:**
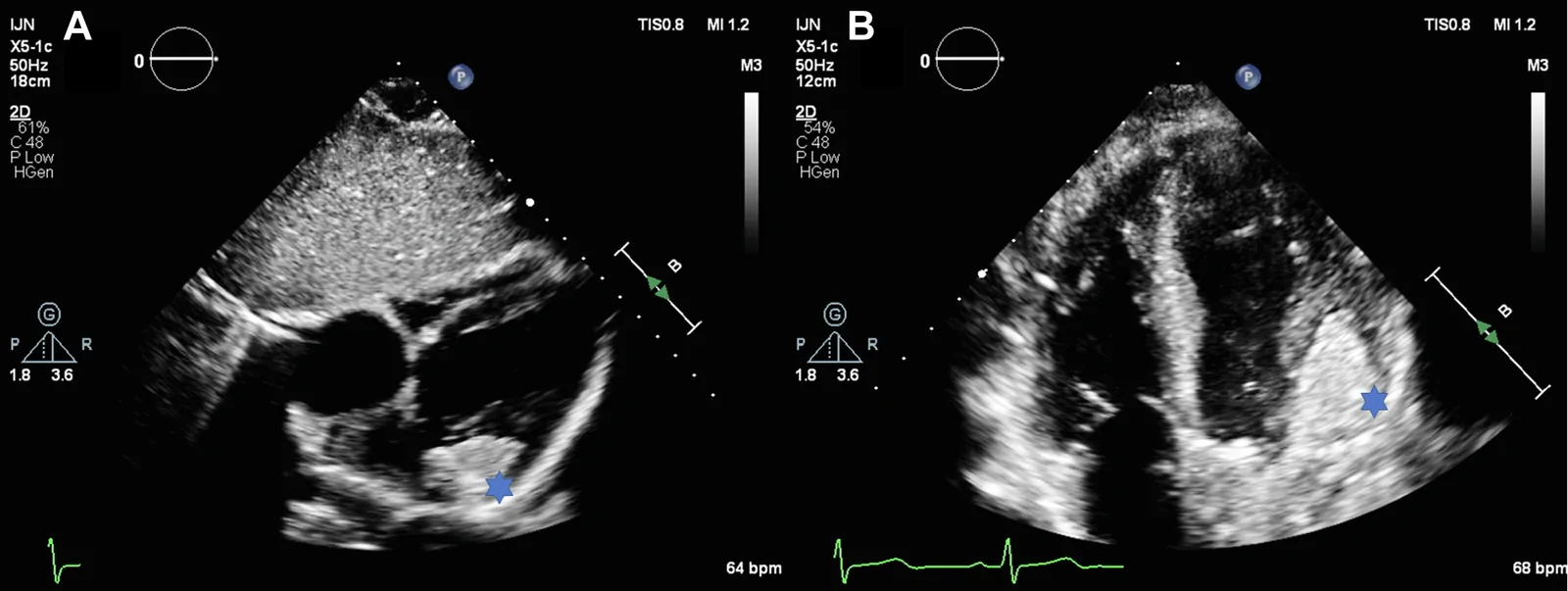
2D Postoperative Transthoracic Echocardiography (TTE) (A) Postoperative transthoracic echocardiogram subcostal view showing residual mass at the left ventricle. (B) Apical four-chamber view showing residual mass. Blue star refers to the residual mass seen on 2D postoperative transthoracic echocardiography. bpm = beats/minute; IJN = internal jugular vein; MI = myocardial infarction; TIS = tissue imaging system.

Histopathological examination demonstrated mature adipocytes admixed with bland spindle cells and collagenous stroma, consistent with a spindle cell lipoma. Postoperatively, the prosthetic valve was stable and functioning well, with only trivial transvalvular leakage and no evidence of obstruction. The postoperative TTE does show residual mass in the LV (Figure 4, Video 8). However, at 1 month, the patient was asymptomatic and resumed normal activities of daily living.

## Discussion

Primary cardiac tumors are rare, with an estimated incidence of 0.001%-0.03% in autopsy series, and approximately 75% are benign in nature.^1^ Among benign cardiac tumors, lipomas are uncommon and may originate from the endocardium, myocardium, pericardium, or, rarely, the cardiac valves. Although often asymptomatic, cardiac lipomas can present with obstructive symptoms, arrhythmias, or embolic phenomena depending on their size and location.^1^, ^2^, ^3^ A spindle cell lipoma is a distinct benign adipocytic neoplasm characterized histologically by an admixture of mature adipocytes, uniform spindle cells, and collagenous stroma, frequently demonstrating CD34 immunopositivity and absence of cytologic atypia.^4^ It is a distinct benign adipocytic neoplasm that typically arises in subcutaneous soft tissues and is exceptionally rare in visceral organs.^4^, ^5^, ^6^ Cardiac involvement is exceedingly uncommon. Recognition of this histologic variant is clinically important as the presence of spindle cell morphology within a cardiac mass may raise concern for malignant neoplasms, which carry significantly different prognostic and therapeutic implications.^7^

A systematic review of the literature was performed in accordance with the 2020 Preferred Reporting Items for Systematic Reviews and Meta-Analyses guidelines.^8^ Searches were conducted in PubMed (MEDLINE) and EMBASE from January 1, 2000, to January 17, 2026 (Supplemental Appendix A). The search yielded 392 records, of which 2 studies met the inclusion criteria with a histologically proven spindle cell lipoma^9^^,^^10^ (Supplementary Figure 1). Table 1 summarizes all reported cases, including this case.

**Table 1:** Summary of Case Report and Reported Cases in Literature

First Author	Age, y/Sex	Cardiac Location	Clinical Presentation	Imaging Findings	Operative Management	Follow-Up
Rathore^9^	64/Female	Aortic valve (left coronary leaflet)	Progressive exertional angina (NYHA functional class II) for 2 y	TTE: mild AS with 2.4 × 2.3-cm echogenic mass; CT confirmed valvular tumor	Tumor excision and mechanical AVR via minimally invasive partial sternotomy	Improved to NYHA functional class I; no recurrence at 1 y
Sauchelli-Faas^10^	54/Female	Pulmonary valve (anterior leaflet)	Acute severe dyspnea; palpitations	TTE/TEE: ∼2.5-cm pedunculated RVOT mass; CT hypodense lesion; MRI T1 hyperintense, T2 hypointense	Tumor excision with pulmonary valve reconstruction (bicuspidization)	No recurrence at 1 y
Soh, 2026	13/Male	Aortic valve, interventricular septum, posterior LV wall	Ejection systolic murmur	Echo: multiple masses involving the aortic valve (2.06 × 1.65 cm), IVS (4.38 × 2.18 cm), and posterior LV wall (5.10 × 3.09 cm), causing severe AR and moderate AS. CMR: lobulated valvular mass (27-28 × 18-20 mm) extending into LVOT; mild T1 hyperintensity, relative T2 isointensity, mild heterogeneous enhancement	Resection of cardiac mass and mechanical AVR with a 23-mm St Jude Regent prosthesis	No immediate postoperative complication

AR = aortic regurgitation; AS = aortic stenosis; AVR = aortic valve replacement; CMR = cardiac magnetic resonance; CT = computed tomography; IVS = interventricular septum; LV = left ventricle; LVOT = left ventricular outflow tract; MRI = magnetic resonance imaging; RVOT = right ventricular outflow tract; TEE = transesophageal echocardiography; TTE = transthoracic echocardiography.

Across the 2 previously reported cases and the present case, several shared features are evident. All lesions arose from cardiac valves, a location that is itself uncommon for primary cardiac tumors, which affect the valves in fewer than 4% of cases.^11^ Clinical presentations were related to hemodynamic disturbance or valvular obstruction, including exertional angina or dyspnea, in contrast to many cardiac lipomas at other sites, which are frequently incidental findings. This highlights that even histologically benign tumors may have clinically significant consequences when located on cardiac valves.^12^

Cardiac imaging played an important role in the diagnostic evaluation but was not definitive in any reported case. Echocardiography reliably identified the presence and dynamic behavior of a valvular mass, whereas cross-sectional imaging helped narrow the differential diagnosis. In the AV case, TTE demonstrated mild aortic stenosis with a 2.4 × 2.3-cm echogenic mass arising from the ventricular aspect of the LCC, which had increased in size on serial imaging. Chest computed tomography confirmed the valvular tumor.^9^ In the pulmonary valve case, TTE and transesophageal echocardiogram demonstrated a well-circumscribed, echodense mass measuring approximately 2.5 cm, pedunculated from the anterior pulmonary valve leaflet and protruding into the right ventricular outflow tract during systole, initially raising concern for thrombus or myxoma. Computed tomography angiography confirmed a hypodense right ventricular outflow tract mass without evidence of pulmonary embolism. Cardiac magnetic resonance imaging revealed a 20-mm pedunculated lesion with slight T1 hyperintensity, slight T2 hypointensity, and moderate progressive gadolinium enhancement, with imaging differentials including papillary fibroelastoma and myxoma.^10^ Similar diagnostic uncertainty has been described for other benign valvular tumors, reinforcing that histopathological examination remains the gold standard for the diagnosis of spindle cell cardiac lipoma.^13^ The characteristic combination of mature adipocytes, bland spindle cells, rope-like collagen, absence of cytologic atypia, and CD34 positivity is critical in establishing the correct diagnosis,^4^^,^^13^ as this has direct management implications, as recognition of spindle cell lipoma supports conservative postoperative surveillance without adjuvant therapy. In all reported cases, complete surgical excision was curative, with no evidence of recurrence at follow-up of up to 1 year.^9^^,^^10^

The present pediatric case adds important new information to the limited literature. First, it demonstrates that a spindle cell cardiac lipoma can occur in childhood, underscoring the need to consider this diagnosis even in younger patients presenting with cardiac masses. Second, it supports existing evidence that surgical management is safe and effective, with excellent short- and medium-term outcomes. Finally, by systematically reviewing all previously reported cases, this report provides the most comprehensive synthesis to date of the clinical presentation, imaging characteristics, operative management, and outcomes of spindle cell cardiac lipomas.

## Conclusions

A spindle cell cardiac lipoma is an exceptionally rare benign tumor. This report describes the first pediatric case and, together with a systematic review of the literature, expands the current understanding of its clinical spectrum. Despite its benign histology, a spindle cell cardiac lipoma may cause significant hemodynamic symptoms related to valvular involvement. Definitive diagnosis relies on histopathological examination, and complete surgical excision appears curative with excellent short-term outcomes.

### Ethics Approval

This study was conducted in accordance with the principles of the Declaration of Helsinki. Institutional review board approval was not required for this single case report and systematic review, in accordance with local institutional policies. The systematic review component involved analysis of previously published data and did not require ethical approval.

### Patient Consent

Written informed consent was obtained from the patient’s parent/legal guardian for publication of this case report.

### Data Statement

The data in this study are included within the article and its supplementary materials. No additional data sets were generated or analyzed beyond those reported. Further details are available from the corresponding author upon reasonable request.

## Funding Support and Author Disclosures

The authors have reported that they have no relationships relevant to the contents of this paper to disclose.

## Appendix

Video 1 Transthoracic Echocardiogram Short-Axis Viewing Showing 2 Masses, One at the Left Coronary Cusp and Noncoronary Cusp, as Well as the Mass at the Left Ventricular Wall

Video 2 Transthoracic Echocardiogram Apical Four-Chamber View Showing Mass at the Left Ventricular Wall

Video 3 Transesophageal Echocardiogram Mid-Esophageal Short-Axis View Demonstrating 2 Masses at the Noncoronary and Left Coronary Cusps and One Mass at the Left Ventricular Wall

Video 4 Three-Dimensional Image from Transesophageal Echocardiogram Showing Left Coronary and Noncoronary Cusp Masses

Video 5 Cardiac Magnetic Resonance Imaging Showing Mass that Encases the Entire Leaflet of the Left Coronary Cusp, Moving Synchronously with Aortic Valve Opening

Video 6 Cardiac Magnetic Resonance Imaging Demonstrating Mass Arising from the Left Ventricular Outflow Tract, adjacent to the Left Coronary Cusp

Video 7 Cardiac Magnetic Resonance Showing Large Lobulated Mass Predominantly Involving the Left Coronary Cusp

Video 8 Transthoracic Echocardiogram Short-Axis View Showing Residual Mass at the Left Ventricular Wall

Video 9 Excision of Cardiac Tumor
