# Supplementary material for: Spindle Cell Cardiac Lipoma in an Adolescent: A Case Report and Systematic Review of Cases
Source: JACC Case Rep. 2026 Jun 19;31(33):108811. doi: 10.1016/j.jaccas.2026.108811 (PMC13523151; doi:10.1016/j.jaccas.2026.108811)
Supplement: Supplementary file 1 [file mmc10.docx]

**Supplemental appendix**

A) Supplementary Figure 1: PRISMA 2020 flow diagram for new systematic reviews which included searches of databases only

**Identification of studies via databases**

Records removed *before screening*:

Duplicate records removed (n = 64)

Records published before year 2000 (n = 30)

Records identified from*:

Total (n=392)

-Medline/Pubmed (n = 212)

-EMBASE (n= 180)

**Identification**

Records screened

(n = 298)

Records excluded**

(n = 106)

Reports sought for retrieval

(n = 192)

Reports not retrieved

(n = 0)

**Screening**

Reports assessed for eligibility

(n = 192)

Reports excluded:

No histological confirmation of Spindle cell lipoma (n = 190)

Studies included in review

(n = 2)

Reports of included studies

(n = 2)

**Included**

B) Search strategy

Database 1: Pubmed/Medline

("cardiac lipoma"[Title/Abstract] OR "heart lipoma"[Title/Abstract] OR "intracardiac lipoma"[Title/Abstract] OR "valvular lipoma"[Title/Abstract] OR "cardiac tumor"[Title/Abstract] OR "cardiac tumour"[Title/Abstract] OR  "benign cardiac tumor"[Title/Abstract]) AND ( lipoma[Title/Abstract])

Database 2: EMBASE

('cardiac lipoma'/exp OR 'heart lipoma' OR 'intracardiac lipoma' OR 'valvular lipoma')

AND (lipoma)
